# The role of mRNA in the development, diagnosis, treatment and prognosis of neural tumors

**DOI:** 10.1186/s12943-021-01341-7

**Published:** 2021-03-05

**Authors:** Yiyang Zheng, Yanyan Luo, Xixi Chen, Huiting Li, Baojun Huang, Baofeng Zhou, Liqing Zhu, Xianhui Kang, Wujun Geng

**Affiliations:** 1 Department of Anesthesiology, The First Affiliated Hospital of Wenzhou Medical University, Wenzhou, 325000 People’s Republic of China; 2 School & Hospital of Stomatology, Wenzhou Medical University, Wenzhou, 325000 People’s Republic of China; 3 Department of clinical laboratory, Wenzhou Medical University, Wenzhou, 325000 People’s Republic of China; 4 Department of Anesthesiology, The First Affiliated Hospital, Zhejiang University School of Medicine, Hangzhou, China

**Keywords:** mRNA, Neural tumor, Tumor therapy, Gene regulation

## Abstract

Neural tumors can generally be divided into central nervous system tumors and peripheral nervous tumors. Because this type of tumor is located in the nerve, even benign tumors are often difficult to remove by surgery. In addition, the majority of neural tumors are malignant, and it is particular the same for the central nervous system tumors. Even treated with the means such as chemotherapy and radiotherapy, they are also difficult to completely cure. In recent years, an increasingly number of studies have focused on the use of mRNA to treat tumors, representing an emerging gene therapy. The use of mRNA can use the expression of some functional proteins for the treatment of genetic disorders or tissue repair, and it can also be applied to immunotherapy through the expression of antigens, antibodies or receptors. Therefore, although these therapies are not fully-fledged enough, they have a broad research prospect. In addition, there are many ways to treat tumors using mRNA vaccines and exosomes carrying mRNA, which have drawn much attention. In this study, we reviewed the current research on the role of mRNA in the development, diagnosis, treatment and prognosis of neural tumors, and examine the future research prospects of mRNA in neural tumors and the opportunities and challenges that will arise in the future application of clinical treatment.

## Background

### Introduction to the current research status of neural tumors

Neural tumors can be divided into two categories: central nervous system tumors and peripheral nervous system tumors, and the formation of this type of tumor is caused mainly due to errors in intrinsic nerve repair after a certain degree of nerve injury [1]. According to the World Health Organization survey report on the incidence and mortality of cancer in 185 countries worldwide in 2018, there were 296,851 new cases of brain and nervous system tumors, with a morbidity rate of 1.6% and a mortality rate of 2.5% among all cancers [2]. The occurrence of neural tumor is a challenge for both patients and doctors, which will not only bring about a decrease in the quality of life and pain of patients, but also cause troubles to surgeons when encountering many neural tumors that are difficult to cure with techniques such as surgery and radiotherapy. At present, a large number of studies have been devoted to elucidating the mechanism of neural tumor formation at the molecular level, and to studying the preventive techniques and therapeutic means for neural tumors.

Central nervous system tumors often occur in the central nervous site, and currently, they are considered to be related with multifactorial pathogenesis, mainly including environmental factors and genetic susceptibility. While central nervous system tumors account for only a small proportion in terms of the overall cancer incidence, curing central nervous system tumors is still a great challenge. The key issues involved are as follows: the correlation of selected targets for specific tumor types, the persistence of operable biomarkers at recurrence, the penetration of blood-brain barrier, and the analysis of primary and acquired drug resistance mechanisms [3]. If methods such as surgery are used for biopsy and treatment, on the one hand, it is easy to damage the relevant central nervous system. On the other hand, because the central nervous system is located deep, it may be difficult for the surgery to touch the site of tumorigenesis. Therefore, there are still many therapeutic limitations at this stage. Further intensified study needs to be done on central nervous system tumors at the molecular level to find the diagnosis and treatment methods at the genetic level, that is, the source of tumorigenesis.

Compared with central nervous system tumor, peripheral nervous tumor is more commonly treated by surgical methods. The underlying cause is not that it is easier to be treated by surgical methods, but that most peripheral nervous tumors (such as malignant schwannoma) are insensitive to chemoradiotherapy, have a poor prognosis and are prone to recurrence after surgery, especially metastases via blood circulation. In addition, the source of peripheral nervous tumor cells is also controversial. Although it was once believed that tumors were generally derived from cancer stem cells, it is currently stated in the literature that peripheral nervous system tumors caused by neurofibromin (Nf1) gene deletion occur in different Schwann cells or restricted progenitor cells closely associated with them, rather than in stem cells [4]. The diagnosis and cure of tumors and the source of tumor cells need to be further studied and explained at the molecular level.

Throughout the nearly 20 years of neuro-oncology research, the rapid development of molecular biology has transformed various fields of basic research and clinical research in neuro-oncology with unprecedented depth and breadth. For example, the superficial RNA sequencing technology in tumors can be used to assess the prognosis of cancer [5]; many regulatory non-coding RNAs can be used as markers for tumor diagnosis and play a role in tumor therapy [6]. Therefore, for neural tumors with the characteristics of proneness to metastasis and difficulty in resection, it is necessary to find a breakthrough in tumor diagnosis and treatment from the perspective of molecular biology.

### Overview of related studies of mRNA in neural tumors

Messager RNA (mRNA) is the only coding RNA in organisms. It is a direct template for carrying genetic information and guiding protein biosynthesis. It connects genetic information in DNA with protein translation and expression, and plays a very critical role in life activities [7]. Through the detection of mRNA level, the expression of genes can be well reflected. Therefore, in the research and development of new drugs for tumors and other diseases, both the upstream DNA of mRNA, the downstream proteins of mRNA, and even the non-coding small RNAs that affect its processing and modification have been fully and comprehensively studied. However, mRNA is sandwiched among them, and its function is relatively simple, so there are few relevant studies, and the breakthrough achievements are mainly focused on several decades ago. However, this is not because mRNA has no research prospect and value. For example, the expression of genes can be well reflected by the detection of mRNA level. In addition, as the “middleman” of gene expression, mRNA has the potential to replace DNA therapy, and the influence on mRNA itself is equivalent to the influence on the genetic information itself to a certain extent.

**Fig. 1:**
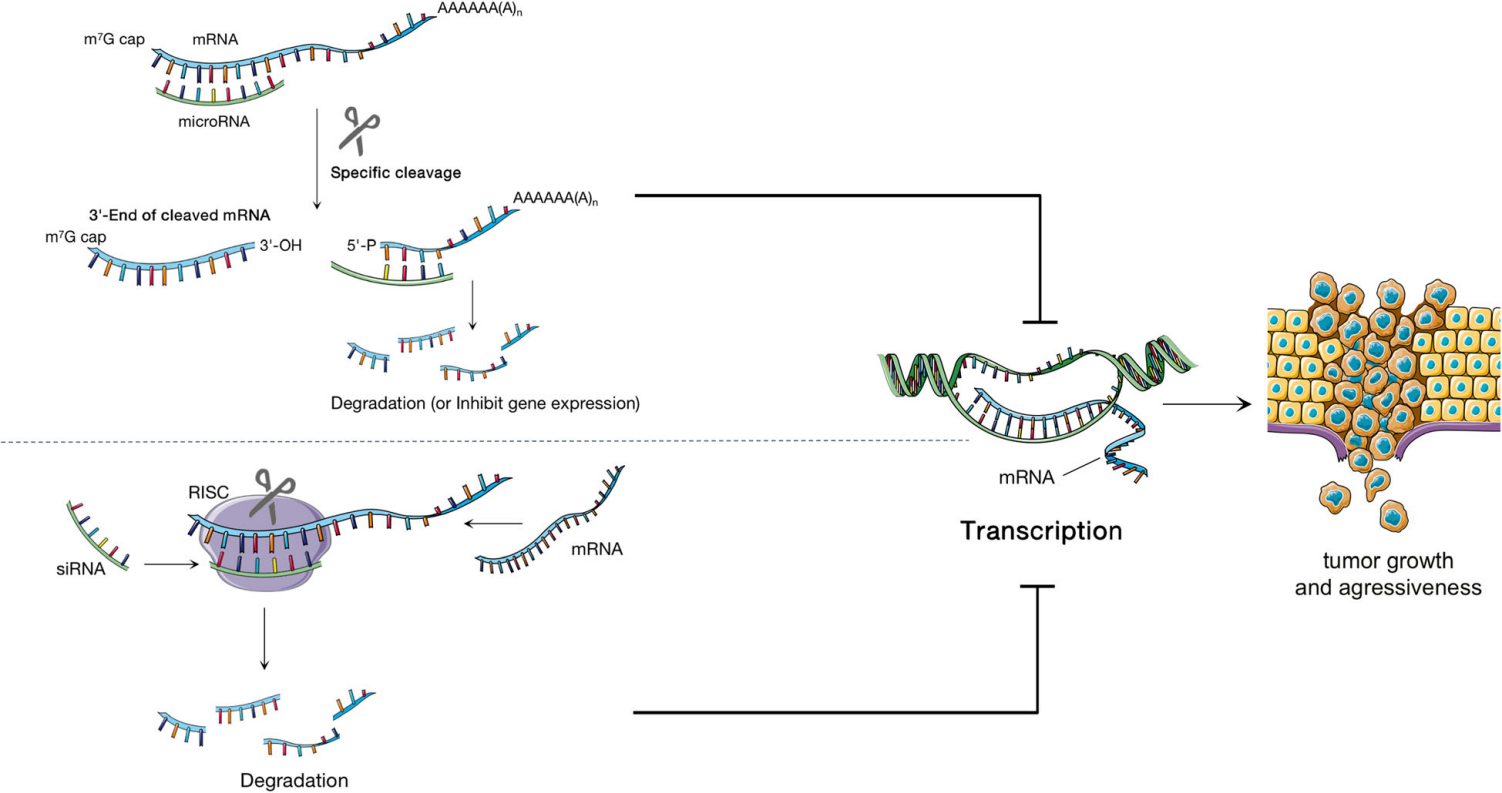
Small non-coding RNA influences tumor development: both miRNA and siRNA can induce the degradation of complementary mRNA in cells, thus preventing the translation of mRNA sequence into protein, thus affecting the growth and metastasis of tumors [11, 12]

In addition, recent studies have shown that the gene expression of mRNAs is also regulated by many non-coding RNAs. Micrornas and mRNA interaction, for example, most of the original view of micrornas can pass and complementary mRNA sequence of bases, the expression of silence after the gene transcription or specificity to inhibit gene expression [8], and now the mainstream view miRNA function mainly through the cost target mRNA stability, rather than by inhibiting their translation work [9]. Synthetic small interfering RNAs (siRNAs) can be introduced into the cytoplasm and induce the specific degradation of mRNAs through RNA interference (RNAi) [10] (Fig. 1).

In the treatment of neural tumors, mRNA can be used as a biomarker and a target for cancer therapy, and the mRNA responsible for encoding tumor antibodies also has the potential to evoke effective anti-tumor immunity [13]. In addition, the mRNA of many proteins can be regulated by other signals, thereby regulating the expression of tumor-related proteins [14], while many tumor cell-derived exosomes also contain related mRNAs that promote cell proliferation. mRNA can produce functional proteins after being transported to recipient cells, which promote the horizontal transfer of genetic information and thus play its key role in the expression of tumor genes. At present, mRNA plays an increasingly important role in the occurrence and development of tumors, treatment and diagnosis, and in recent years, mRNA tumor vaccines have been gradually applied to cancer treatment [15]; in addition, mRNA therapy has also been beginning to play a role in the treatment of various types of tumors as an emerging gene therapy.

## mRNA plays a role in different central nervous system tumors

We referenced the classification method of central nervous system tumors by the World Health Organization in 2016 [16, 17], learned the malignant degree and related research progress of various types of central nervous system tumors, selected the following categories of tumors and summarized the relevant progress of basic research and clinical research of mRNA in these tumors.

### Diffuse astrocytic and oligodendroglial tumors

According to the International Classification of Diseases (ICD-O) for morphologically encoded neoplastic diseases, all tumor numbers in this category are number 3 (i.e., malignant tumors), and according to the classification of IDH wild-type (IDH-wt) and IDH mutant type (IDH-mt) as well as 1p/19q deletion [17], in general, diffuse astrocytic and oligodendroglial tumors are mainly treated with surgery, chemotherapy, and radiotherapy at this stage, but the tumor may recur after surgical resection, and some of the recurrent tumors are more malignant than the primary tumors [18].

**Fig. 2:**
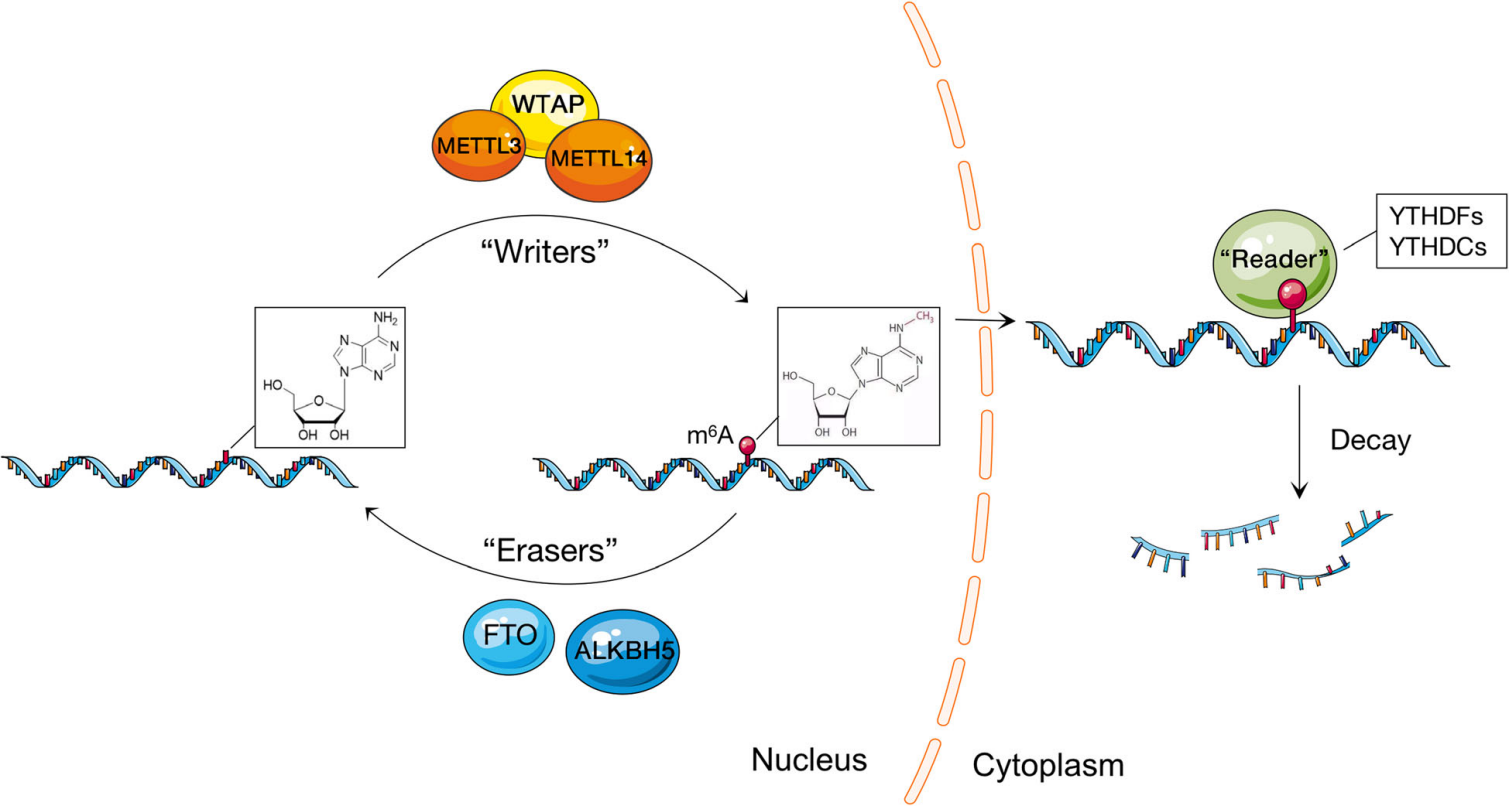
The m6A methylation modification process of mRNA: METTL3, WTAP, METTL14 and other methyltransferases act as “writers”, making the m6A site of mRNA methylated. FTO and ALKBH5 acted as demethylase, and their effects were opposite to those of methyltransferase. In addition, there is a class of m6A recognition and binding proteins, mainly including YTHDFs and YTHDCs, which can bind to this methylation site to induce mRNA decay, thus affecting protein synthesis and the occurrence and development of related tumors

Glioblastoma (GBM) is the most malignant glioma among astrocytic tumors, and primary GBM is the most common type. Its most common gene mutation site is the promoter region of the telomerase reverse transcriptase gene (TERT; OMIM 187270), and TERT promoter mutations are associated with high mRNA expression levels [19]. Alternatively, methylation modification of mRNAs has assumed an important position in glioblastoma studies, but its specific mechanism of action remains unelucidated. Methylation modification of mRNA is a mode of cellular gene regulation, of which m6A (N6-methyladenosine, m6A) modification for the treatment of tumors has been a high-profile studies topic in recent years. And at present, the study on the modification of M6A mRNA is the most thorough, which is attributed to the emergence of M6A-seq, MERIP-seq, Scarlet, LAIC-seq and other analytical technologies [20]. M6A modification is a process in which methyltransferases catalyze the methylation modification of adenine at the 6 N position, and three types of enzymes are generally involved in this process: methyltransferases, demethylases, as well as m6A recognition proteins. METTL3 is a methyltransferase, and glioblastoma stem cells have high levels of METTL3 with global m6A modification [21]. In some cases, the loss of METTL3 leads to a reduction in M6a, which can induce differentiation [20]. In addition, ALKBH5, a demethylase of m6A, can regulate the proliferation and self-renewal of glioblastoma stem-like cells by regulating the stability of pre-mRNA and the expression of FOXM1 gene [22]; FTO, a demethylase of another m6A, is also considered to have the potential to be a new target for glioblastoma therapy [23]. Studies have shown that removal of FTO in vitro can increase the level of m6A, reduce cell proliferation and colony formation, and increase cell apoptosis, which is consistent with the role of m6A in cell differentiation, and the absence of FTO enhances the cell differentiation induced by all-trans retinoic acid [24]. In addition, oncometabolite(R) -2-hydroxyglutaric acid has been shown to inhibit FTO enzyme activity, resulting in anti-tumor effects in vitro and in vivo [25]. Regarding m6A-binding proteins, there are two common subtypes, YTHDFs and YTHDCs. Han D et al. found that long-lasting neoantigen-specific immunity is a demethylase regulated by its methylation by m6A on mRNA through the m6A-binding protein YTHDF15. Loss of YTHDF1 in classical dendritic cells (cDCs) in vivo enhances the cross-presentation of tumor antigens and the cross-priming of CD8 + T cells [26]. In addition, YTHDF2 links RNA epitopic transcription modification to GSC growth, laying the foundation for the specificity of YTHDF2-Myc-IGFBP3 axis as a new therapeutic target for glioblastoma [27]. Therefore, m6A methylation modification of mRNAs is expected to be more intensively studied in neural tumor therapy. (Fig. 2).

Of course, in addition to mRNA methylation, there are some studies on mRNA in GBM, for example, magnetic induction therapy using magnetic nanoparticles as magnetothermal media can specifically treat tumors, and magnetic nanoparticles can carry different chemotherapeutic drugs and connect antibodies and genes. This treatment modality is efficacious in many aspects. In a glioblastoma model, the injection of nanoparticle-formed mRNA encoding interferon regulatory factor 5 and combined with its activating kinase IKKβ can reverse the immunosuppression of tumor-associated macrophages (TAMs) and reprogram them into a phenotype that induces anti-tumor immunity and can promote tumor regression [28]. In addition, it has been found that a specific hypoxic correlation of mRNA expression in glioblastoma multiforme, and the mRNA expression levels of hypoxia-inducible genes and stem cell genes can be used as important tumor markers in glioblastoma multiforme patients [29].

Diffuse astrocytomas, particularly wild-type (IDH-wt) astrocytomas, often present with EGFR and PTEN gene mutations, as well as chromosome 7 polysomy, loss of heterozygosity on chromosome 10q, and TERTp gene mutations. The expression levels of some protein mRNAs are of reference significance in evaluating the malignancy and prognosis of tumors. For example, the expression level and methylation of AREG in cancer tissues depend on the grade of astrocytoma [30]; insulin-like growth factor 2 mRNA-binding protein 3 (IGF2BP3) can predict the poor prognosis of high-grade astrocytoma [31]; CHI3L1 mRNA expression can be used as a biomarker for the prognosis of glioma patients [32]. In addition, in diffuse astroglioma, there is an interaction between miRNAs and mRNAs, and Moser Joanna J et al. observed that three miR34a-targeted mRNAs and two miR-195-targeted mRNAs were down-regulated, while one miR-195-targeted mRNA was up-regulated, demonstrating the differential regulation of mRNAs by specific miRNAs [33].

### Meningiomas

Meningioma is one of the most common primary tumors of the brain and is divided into three stages according to WHO stage: I, II, and III. Most meningiomas present only as benign tumors in stage I, while meningiomas are highly recurrent and fatal when they progress to stages II and III. Most patients with meningioma have no obvious clinical symptoms in the early stage. With the development of the disease, the tumor invades the brain tissue, resulting in further increase of intracranial pressure, causing further symptom changes, such as headache, epilepsy, and leading to a series of related symptoms according to the size of the tumor and the site of invasion [34]. At present, surgical treatment is the most effective means of treating meningioma. Due to the special location of the tumorigenesis, when total resection of the tumor is not possible, partial radiotherapy can be performed in combination. Radiation and sex hormones are considered to be one of the causes of meningioma, but they have not yet been clearly confirmed. At present, it can be clarified that NF2 gene mutation located on chromosome 22 will lead to the occurrence of meningioma [35]. In addition, through the analysis of familial genes of meningioma pathogenesis, it is found that there are a few genes that possess meningioma susceptibility but do not have NF2 gene mutations in the family [36], so in addition to NF2, there should be the presence of other mutated genes.

mRNA can be used as an important tool for the exploration of potential molecules and related pathways in the development process of meningioma. For example, through biological information technology analysis, it can be found that the levels of 56 mRNAs were increased, and the expression of 179 genes was down-regulated in meningioma patients [37], which provides a direction for the investigation on the pathogenesis and further development of meningioma. Through sample analysis of meningioma patients, it can be found that the expression of EMP2 mRNA is much higher in meningioma patients than in non-meningioma patients, making it as a potential marker for further screening and diagnosis of meningioma [38]. From a proteomic perspective, the process of mRNA processing is closely related to the high recurrence rate of meningioma, and patients with rapidly recurrent meningioma have a unique protein pattern of focusing on mRNA processing [39].

### Ependymal tumors

Ependymoma is the third most common central nervous system tumor in children. The clinical manifestations of children are paroxysmal vomiting, nausea, and headache, and the recurrence rate after surgical resection is very high. Children with ependymoma have a high mortality rate and ventricular aneurysm is resistant to conventional treatment. A previous study in genomics and transcriptomics can further confirm potential therapeutic targets for ependymomas [40]. At present, for grade I and II ependymomas, radical surgical treatment is generally possible, but for grade III ependymomas, due to their high degree of malignancy, conventional treatment can only be used as palliative treatment. Therefore, it is necessary to develop new treatment methods for the more malignant and difficult to resectable ependyma.

Although ependymomas have different molecular definitions and classifications, there is actually a balanced set of genomes for ependymomas, which makes it very difficult to predict the prognosis of ependymomas. But it can be analyzed by mRNA-miRNA network and through applying weighted co-expression network analysis (WGCNA), so as to collect seven pairs of key genes in the co-expressed gene network: CYP11B1, KRT33B, RUNx1t1, SIK1, MAP 3 K4, MLANA and SFRP5 that are regulated by miR-15 and miR-24-1. These 7 pairs of miRNA-mRNA play a key role in the growth and inhibition of ependymomas [41]. However, we believe that at this stage, the above findings can only be used as a reference, that is, the future studies suggest a direction, how to intervene in these genes, regulate their expression, and develop new therapeutic methods according to this method, these problems have not been solved, so gene therapy is not mature in this field. In addition, because the recurrence rate of ependymoma after surgical resection is high, it is urgent to find a good prognostic indicator. Telomerase is widely exist in the human body cell, many scientists think that the activity of telomerase in the past strongly associated with people’s lifespan, but want to change the telomerase activity and change the life of the related research all ended in failure, the reasons include: the activation of telomerase was shown to cause cancer. Based on this, a study has found that telomerase activation is closely related to the prognosis of ependymoma by measuring telomerase activity and telomerase reverse transcriptase (hTERT) mRNA expression [42]. This suggests that although it is difficult to alter telomerase itself to affect human lifespan or the occurrence and development of cancer cells, it may be able to be used as a prognostic indicator.

### Pineal region tumor

The pineal gland is a small secretory gland located in the brain, and the main cells of the pineal gland are pineal parenchymal cells and glial cells, which secrete melatonin, receive light and dark cycle information in the environment, and promote feedback from the central nervous system. Pineal region tumor compression can lead to headache, invasion of the hypothalamus leading to diabetes insipidus and other hypothalamic neuropsychiatric symptoms.

The characteristics of pineal tumor cells in vitro have been confirmed by culturing pineal tumor cells in vitro and measuring the mRNA expression of protein genes such as TPH, AANAT, and HIOMT [43]. At the same time, there are also suspicious genes for pineal region tumors screened out by microarray technology, and then the level of mRNA is detected, so that it further serves as a prognostic marker as well as provides the direction of research on tumor development mechanism by confirming the genes with differential expression [44]. For example, the mRNA of HIOMT is widely expressed in pineoblastoma as well as pineocytoma, which indicates that HIOMT shows great promise as a marker for the diagnosis of pineal tumors [45].

Cellulogenesis and molecular analysis of pineal tumors have been increasingly used for further specification of different GCT subtypes [46]. Schneider et al. showed in their study that central nervous system GCTs had frequent aberrations in CCND2 (12p13), RB1 (13q14) and PRDM14 (8q13) genes, which play a role in the cyclin/CDK-RB-E2F pathway involved in transcriptional regulation of primordial germ cell specification as well as in the development of CNS GCTs [47, 48]. Therefore, the mRNAs expressed by related genes and the related proteins translated by them are also expected to be possible therapeutic targets.

### Germ cell tumors

Central nervous system germ cell tumors refer to a heterogeneous type of tumors that are formed by the transformation of primordial germ cells or pluripotent germ cells. Germ cell tumors can occur at any primordial site of normal or ectopic migration, can be primary beyond the ovary and testis, and can also occur beyond the gonads, such as the pineal gland (predominant), sacrococcygeal vertebrae, mediastinal cavity, and retroperitoneal cavity, which are still poorly known. In this review, we focused on intracranial germomas, namely, pineal, suprasellar, and basal ganglia germomas. Studies have shown that intracranial germ cell tumors may develop through two different pathogenesis: one is KIT/RAS changes, increased KIT mRNA expression and severe chromosomal instability, and the other is the absence of any of the above abnormal unknown mechanisms, while the presence of KIT/RAS changes is usually associated with the upregulation of KIT mRNA and chromosomal instability. Although the above studies only analyzed the big data of cases, and there is no exact experiment to clarify the mechanism of modification at this stage, it suggests that we can link the expression level of KIT mRNA with the pathogenesis of intracranial germ cell tumors [49].

Over the past few years, more than 140 RNA modifications have been identified, as well as their upstream and downstream related roles in regulating the fate of target RNAs, namely, the roles in stability, translation and splicing [50–54]. Recently, the content of m6A in mRNA has been considered to be related with cancer initiation and progression. Among the TGCT subtypes, the abundance of m6A and the expression of VIRMA/YTHDF3 vary, with higher levels in SE, suggesting a contribution to the maintenance of the SE phenotype. VIRMA and YTHDF3 may collaborate in the m6 A modification mechanism in TGCT, and their transcript levels can be used to accurately distinguish SE from NST, constituting a novel candidate biomarker for patient management. These markers involved may also constitute therapeutic targets and therefore represent potential cancer biomarkers [55].

In addition, a recent study has shown [56] that the identification of functional miRNA-mRNA interactions in cancer (i.e., the interactions that alter gene expression in cancer cells) can help delineate post-regulatory mechanisms and may lead to new therapies to control cancer progression. First find the genes associated with the disease and then find the miRNAs that target these genes, that is, miRNA-mRNA interactions. The sequence-based prediction of the interactions are refined based on two well-known methods for learning miRNA-mRNA interactions, namely TaLasso and GenMiR++. miRNA-mRNA interactions may be a potential target for future laboratory experiments to identify TGCT-specific interactions and develop new therapies.

### Lymphoma

Central nervous system lymphomas include primary central nervous system lymphomas and secondary lymphomas with systemic lymphomas invading the central nervous system. The incidence of the disease is low, accounting for 1 to 3% of central nervous system tumors. With the use of immunosuppressive agents, the incidence of the disease has been increasing in recent years. Primary lymphoma of the central nervous system accounts for about 8%, about 50% of intracranial lymphoma cases are associated with systemic lymphoma, and central nervous system lymphoma can develop at any age. In addition, the clinical manifestations of malignant lymphoma not only share certain common characteristics, but also have great differences according to different pathological types, invasion sites and extents.

mRNAs can be functionally transferred between cells by exosomes and it is called “exosome shuttle RNA (esRNA)”. mRNA in plasma exons has been shown to be a potential liquid biopsy method. It has been shown that exosome mRNAs can exert anticancer effects by repressing genes associated with tumor development [57, 58]. On the one hand, siRNAs, miRNAs, mRNAs and lncRNAs with tumor suppressor activity can be loaded into exons and transferred to target cells to exert anti-lymphoma effects. On the other hand, we can interrupt the process of RNA internalization into exons, especially for those with cancer metastasis and drug resistance, and this may be used as a potential therapeutic means against lymphoma. In addition, lymphoma-derived exons can also reprogram the bone marrow environment and lead to tumor progression. Adult T-cell lymphoma/leukemia (ATLL) cell exons can transfer TaxmRNA to mesenchymal stem cells (MSC), activate the NF-κB pathway, and promote tumor proliferation, progression, and angiogenesis [59].

In addition, the integrity, diversity and abundance of cellular protein products are largely controlled by post-transcriptional regulation, which includes many intermediate steps between transcription and translation. This highly coordinated process: new RNAs undergo variable splicing, editing, polyadenylation, capping, 3′ end formation and nucleocytoplasmic transport to the ribosome before translation and final degradation [60]. Post-transcriptional regulation is primarily controlled by RNA-binding proteins (RBPs) and small RNAs that bind primarily to specific elements located in the untranslated region (UTR) of target mRNAs. It has been confirmed that HuR and eIF4E collaborate to maintain tumorigenesis because HuR promotes eIF4E expression by increasing the stability of its transcripts. In turn, eIF4E stimulates translation of cancer-inducing targets, which are also stabilized by HuR [61].

Translation of mRNAs with extensive 5′ secondary structures is poor, so binding of eIF4E to the cap as well as the role of eIF4A is essential for their translation [62]. Since eIF4E is a component that directly binds to the 5′ cap, most studies have focused on this key component and its regulation. Many data suggest that eIF4F, a translation initiation factor composed of eIF4E, eIF4A and eIF4G, plays an important role in maintaining the translation of a variety of important leukemia and lymphoma oncogenes and transcription factors. Phosphorylation of 4EBP1 through mTOR can release eIF4E; therefore, eIF4E is a tempting target for therapeutic inhibition, and some researchers have used the antiviral drug ribavirin to mimic the cap structure to allow its eIF4E to bind to mRNA and change mRNA stability, which in turn achieves the purpose of targeted therapy [63].

### Embryonal tumors

Embryonal tumors include malignant germ cell tumors, neuroblastomas, and primitive neuroectodermal tumors. It predominates in children and adolescents. Gene amplification in tumors often results in increased mRNA expression of the amplified loci. In central nervous system embryonal tumors, Xing Fan et al. [64] found a significant positive correlation between increased gene dose and hTERT information level, with hTERT amplified in recurrent tumors but not in primary foci, indicating that this locus may be associated with tumor progression. The data suggest that hTERT gene amplification is relatively prevalent in embryonic brain tumors, while increased hTERT mRNA expression may be associated with biologically aggressive tumor behavior.

Among embryonal tumors, medulloblastoma (MB) is the main tumor. MB is the most common brain tumor and is a pathology composed of four molecules. Despite multimodal therapy, 30% of patients eventually relapse and develop fatal metastases within 5 years. The main contributors to metastatic spread are lymphatic growth factors, VEGFC and their receptors/co-receptors. Manon Penco-Campillo et al. [65] studied the visualization of VEGFR3 and PROX1 in the cerebellum, located in Purkinje cells and the outer granular layer, respectively, and both mRNAs regulate neuronal development in the early postpartum period. Therefore, high levels of VEGFR3 and PROX1 mRNA may be associated not only with lymphatic vessels, but also with neuronal development. In the study by Yu J. et al. [66], RMB5 was shown to inhibit glioma development by inhibiting Wnt/β-catenin signaling, and the Wnt/β-catenin signaling pathway could promote cell proliferation and migration in MB.

A recent study has indicated that MB shows frequent epigenetic alternations, so Anqi Xiong et al. [67] treated MB cell lines with drugs that inhibited DNA methylation or histone deacetylation and found in their study that NRBP2 mRNA expression was up-regulated, indicating that it is under epigenetic regulation in cultured MB cells. In addition, forced overexpression of NRBP2 in MB cell lines results in a dramatic reduction in cell number, increased cell death, impaired cell migration and inhibition of cell invasion in vitro. Taken together, downregulation of NRBP2 may be a feature of MB cells that escape growth regulation.

In addition, in MB samples from the same patients, Vinod Kumar et al. [68] performed miRNA analysis, RNA sequencing and intelligent path analysis to study the pathogenesis of MB using the miRNA-mRNA profiling mentioned above, and found that manipulating miR-217 may have therapeutic potential for MB patients.

### Other CNS tumors

#### Melanoma

Melanoma, usually defined as malignant melanoma, is a highly malignant tumor of melanocytic origin that occurs mostly in the skin, but also in the mucosa and viscera, accounting for approximately 3% of all tumors. Cutaneous malignant melanoma accounts for the third most common cutaneous malignancy (about 6.8 to 20%). In recent years, the incidence and mortality of malignant melanoma have been increasing year by year, and its age of death is lower compared with other solid tumors. In addition to early surgical resection, malignant melanoma lacks specific treatment and has a poor prognosis. Therefore, early diagnosis and treatment of malignant melanoma is extremely important.

It has been shown that, the expression of p62 of the mRNA found in TCGA melanoma, and the factors of NF-κB signaling, including NFKB1, RELA, or MELK, the stability of mTOR-related genes such as S6K1, the cytoprotective agent NRF2, or the transcription factor MYC as well as mRNAs encoding FERMT2 and other pro-metastatic factors, can affect the process of melanoma development and can be used as important therapeutic targets in the future [69]. However, the emerging molecular mechanism is also applicable to melanoma that the N 6-methyladenosine (m6A) RNA methylation that regulates gene expression at the post-transcriptional level mentioned above can chemically modify the messenger RNA (mRNA) and non-coding RNA of eukaryotic cells. Seungwon Yang et al. [70] have demonstrated that the m6A demethylase FTO regulates melanoma growth and mediates melanoma resistance to anti-PD-1 antibodies in vitro and in vivo. They identified specific FTO-mediated and m6A-mediated mechanisms that contribute to the development of melanoma and resistance to anti-PD-1 blockade, and they also demonstrated that the combination of FTO inhibition and anti-PD-1 blockade reduced resistance and improved anti-melanoma responses.

#### Hemangioma

Hemangioma is a congenital benign tumor or vascular malformation commonly seen in the skin and soft tissues formed by the proliferation of angioblasts during the embryonic period, and it is more common in infants at or shortly after birth. Commonly used treatments are: surgical resection, radiotherapy, cryosurgery, sclerosing agent injection and laser irradiation. It has been shown [71] that the expression of some mRNAs can act on the corresponding receptors to affect the pathogenesis of hemangiomas. However, it has also been demonstrated that the miRNA-mRNA interaction above can serve as a expression profile for comprehensive analysis [72]. However, in addition to this, it is exciting that mRNA levels can also be used as a distinguishing criterion for hemangioma-related diseases. A relevant study by the Jochen Rössler team [73] demonstrated that β1 – adrenergic receptor mRNA levels reveal the distinction between infantile hemangiomas and vascular malformations. IH is the most common benign tumor in childhood and exhibits specific biological features, but no mature model of IH has yet been established and the underlying molecular mechanisms cannot be fully determined. Endothelial cells of hemangiomas, IH, VM, and LM, can be distinguished by their differences in β-adrenoceptor subtype mRNA expression, particularly when they can be used to distinguished the vascular malformations composed of veins, arteries, or lymphatic vessels. It happens that there is a similar study [72] showing that VEGF-R1 mRNA levels reveal the distinction and similarities between congenital and usual infantile hemangiomas. Congenital tumors can be divided into rapidly involuting congenital hemangiomas (RICH) and non-involuting congenital hemangiomas (NICH), both of which are similar in appearance, location, and size, and have some overlapping histological features with infantile hemangiomas. At the molecular level, none of them express glucose transporter-1, a diagnostic marker for infantile hemangiomas. Arnaud Picard et al. proposed [74] that the relative mRNA expression levels of IGF-2 and membrane-associated FLT-1 provide molecular evidence linking RICH and NICH and distinguishing RICH and NICH from common infantile hemangiomas. The expression level of mRNA is well used as a distinguishing and diagnostic marker for hemangioma.

## mRNA in peripheral nervous tumors

Peripheral nervous system tumors, defined as tumors of all neural structures occurring beyond the brain and spinal cord, belong to the large category of soft tissue tumors and are closely related to plastic surgery, dermatology, orthopedics and neurosurgery [75]. Common tumors of the peripheral nervous system include schwannoma and neurofibroma, which are distinguished by cellular components. Neurilemmoma, also called nerve sheath tumor, are composed of nerve sheath cells and are often accompanied by intraneural or extraneural lesions. The cellular composition of neurofibromas is more complex than that of schwannomas, including Schwann cells, fibroblasts, perineural fibroblasts, axons, and endothelial cells [76].

Although schwannoma is a benign tumor, it may lead to a sharp decline in the living standards of patients. The location of schwannoma and the nerves involved may cause pain, sensory loss or abnormality, limb weakness and other symptoms. The treatment is mainly surgery and radiotherapy. The surgical options depend on the location of the growth of the schwannoma. If common facial schwannoma causes facial dysfunction, surgery can be performed through labyrinth, craniotomy, or suboccipital craniotomy. If vestibular schwannomas and lesions involving the mastoid process, geniculate ganglion, and tympanic membrane can be operated via the transcranial middle fossa or transmastoid approach to preserve partial vestibular and hearing function. For some patients, it is also possible to use nerve decompression to relieve facial nerve dysfunction and prevent further deterioration of neurological function. For patients who do not require or cannot undergo surgical intervention, radiotherapy still carries the risks of uncontrollable tumors, worsening facial function, hearing loss and further tumor deterioration [77]. For gastrointestinal schwannoma, on the other hand, it is usually a benign tumor, and after taking into account imaging studies and preoperative biopsy, complete surgical resection is generally selected, and adjuvant chemotherapy and radiotherapy are less commonly used [78]. There is also a precedent for medical treatment of schwannoma, and bevacizumab treatment with anti-VEGF monoclonal antibodies, but it can be considered only when the patient’s condition is stable and there are more serious side effects [79].

Neurofibromas are divided into two subtypes, NF1 and NF2, of which NF1 is caused by mutations in the NF1 gene, is an autosomal dominant disorder that can affect many systems and is the most common neurofibroma. Common clinical symptoms of NF1 type are symptoms such as café-au-lait macules, cutaneous neurofibromas, and Lisch nodules. Generally, NF1 neurofibromas do not require special treatment, and CO2 laser removal or surgical removal can be considered if symptoms such as pain or itching occur or cause changes in appearance. In the case of multiple neurofibromas and in the advanced stage or at moderate to high risk of transformation, an investigational agent may be considered along with resection of the symptomatic lesion. If there is a suspected risk of cancer, multidisciplinary intervention should be performed, and multiple treatment methods including chemotherapy, radiotherapy, surgery and clinical trials should be appropriately adopted [80].

At present, the oncogenesis mechanism and diagnosis of tumors have been extended to genetic and molecular pathways. By comparing with normal tissues and pathological tissues, mRNA and miRNA with differences in expression can be identified, and mRNA and protein-protein interaction network (PPI) can be constructed to explain the function of mRNA and miRNA. MiRNAs are novel non-coding RNA molecules that are key molecules in the post-transcriptional regulation of the expression of a variety of genes, and they are widespread in biological processes such as tumors [81, 82]. Therefore, understanding the role of mRNA on tumors is of great significance in two major peripheral nervous tumors: schwannoma and neurofibroma.

### Schwannoma

Ahmed SG et al. found that caspase recruitment domain (ASC) mRNA was decreased in schwannoma while exhibiting ASC methylation. Targeted delivery of hASC and mASC with adenovirus-associated vector (AAV) plays a tumor therapeutic role through the apoptotic pathway without damaging neurons, suggesting that ASC may prevent the development of schwannoma by inducing apoptosis and cell cycle arrest [83]. In the study by Sohn EJ et al., it was found that curcumin could induce apoptosis of schwannoma cells through the upregulation of miRNA 344A-3p. In the study performed by Lei Y et al. [84], ISG15 mRNA and protein expression in vestibular schwannomas was markedly upregulated, and was a central gene of the PPI network of schwannomas. Alternatively, transcriptional co-pressor (TLE1) is also one of the central nerves of the PPI network. PRICKLE1 mRNA is significantly down-regulated in vestibular schwannomas, while GALR1 is abundant in neuroactive ligand-receptor interactions, and both PRICKLE1 and GALR1 are targets of hsa-miR-30c-5p and hsa-miR-30a-5p, suggesting that hsa-miR-30 may play a key role in vestibular schwannomas [85]. In sporadic vestibular schwannomas, Erbb2-interacting protein (Erbin) gene was found to be up-regulated, while Erbin was able to participate in tumor production through neuregulin 1 (Nrg1) activation of pathways such as PI3K/AKT. In addition, PDGFC and PI3K genes were also up-regulated, both of which are involved in cell proliferation and growth [86]. Members of the metalloproteinase protein (ADAM) family are therapeutic targets in many solid tumors, ADAM9 mRNA expression is 8.8-fold higher in vestibular schwannomas compared with normal tissues, and the expression level of ADAM9 is significantly correlated with hearing loss in patients and may be a marker for judging tumor growth and invasion [87].

### Neurofibroma

Neurofibromatosis type NF1 has been found to be associated with gene mutations, and NF1 gene mRNA levels of specific subtypes are closely related to the severity of neurofibromatosis type 1 [88]. Some patients with neurofibromatosis type NF2 also present with vestibular schwannoma. This tumor is associated with NF2 gene deletion on chromosome 22 and patients are also more likely to develop into more tumors, including ventriculoma, ependymomas, and bilateral vestibular schwannomas [89] (Table 1).

**Table 1:** mRNA species in different types of cancer

Cancer Type	mRNA Species	Mechanism	Function	Time (year)	Refs
Glioblastoma	EGFRvIII mRNA	Unknown	Diagnosis biomarkers	2008	[90]
	AMACR mRNA	Unknown	Diagnosis biomarkers	2020	[91]
	IGFBP2 mRNA	The occurrence of IDH1 mutation, Hsp27 expression and TERT promoter mutation	Prognostic biomarker	2013	[92]
	CHI3L1 mRNA	Acts on stem cells and drive the formation of tumor vascularization	Diagnosis biomarkers	2019	[93]
Meningioma	EMP2 mRNA	Unknown	molecular marker	2020	[38]
	hTERT mRNA	Changes in the splicing pattern of hTERT splice variants (α-deletion and β-deletion)	prognostic biomarkers	2019	[94]
	PTEN mRNA	Unknown	Low expression is used as a marker to predict malignancy	2019	[95]
	Ki-67 mRNA	Unknown	High expression is used as a marker to predict malignancy	2019	[95]
Pineal region tumor	c-myc mRNA	Drives cell growth by blocking the expression of some genes associated with DNA packaging and cell death	Play a role in oncogenic processes of cancer	2008	[43, 96]
Embryonal tumors	hTERT mRNA	Biologically aggressive tumor behavior	High expression is used as a marker to predict malignancy	2013	[64]
Schwannoma	ASC mRNA	Induce apoptosis and cell cycle stagnation	Promote angiogenesis	2019	[83]
	ISG15 mRNA	Unknown	Diagnosis biomarkers	2019	[85]
	PRICKLE1 mRNA	Unknown	Diagnosis biomarkers	2019	[85]
	Erbin mRNA	Activation of the PI3K/AKT pathway	Cell proliferation	2017	[86]
	ADAM9 mRNA	Unknown	Diagnosis biomarkers	2020	[87]
Medulloblastoma	VEGFR3 mRNA & PROX1 mRNA	Unknown	Regulate neuronal development & Associate with the spread of the tumor	2020	[65]

## Exosome mRNA in (neuro) tumor therapy

Since mRNAs have no risk of genomic integration and the encoded proteins do not need to cross the nuclear envelope to be expressed in the cytoplasm, the interest in using mRNA as a therapeutic tool to treat tumors has greatly been increasing. However, due to the fact that mRNA is not stable enough to easily cross the hydrophobic lipid membrane of cells and is easily degraded outside cells, the effective and safe delivery of treatment-related mRNA remains a major challenge for its wide application in clinical treatment [97]. Therefore, an effective delivery system is urgently needed to overcome this limitation. Over the years, a variety of vectors for in vivo accounting delivery have been constructed — viral vectors (e.g., adeno-associated viral vectors) [98, 99], nanoparticles (e.g., lipid- and lipid-derived nanoparticles, polymer-based nanoparticles) for mRNA delivery [100, 101], but these strategies have potential concerns such as toxicity, immunogenicity, manufacturing costs, and inability to cross specialized physiological barriers such as the blood-brain barrier.

Exosomes are naturally transported nanovesicles secreted by a variety of cell types and contain cell surface compounds that enable them to penetrate various biological barriers (e.g., the blood-brain barrier) [102]. In addition, exosomes can avoid diffusion into tissues by monocytes/phagocytes by directly stimulating target cells and transferring membrane receptors between cells [102], while exosomes have excellent stability in vivo and have lower cytotoxicity than synthetic delivery carriers [103]. These natural properties have considerable advantages in using exosomes as ideal delivery systems for gene therapy.

Exosomes can transmit not only soluble proteins, but also a variety of coding or non-coding RNAs that alter gene expression in recipient cells, thus constituting an excellent communication system between cells [104]; in addition, they are involved in the prediction, progression and treatment of various diseases. For example, in the immune system, the exosome exerts the function of differential expression of related gene mRNAs to regulate the function of immune cells and induce immunoregulatory mechanisms [105]; in transplantation surgery, the exosome mRNA can predict the risk of rejection [106]; in IgA nephropathy, the exosome CCL2 mRNA can be used as a biomarker reflecting the active damage of IgA renal tissue and the deterioration of renal function [107]; the exosome HBx mRNA can affect the hepatitis B liver microenvironment [108]; in tumors, the exosome can carry mRNA as a potential tumor biomarker [109], and it can also carry functional mRNA to inhibit genes related to inflammation, cell proliferation and angiogenesis so as to inhibit tumor growth [110].

In neural tumors, different expression profiles of exosome mRNA can distinguish patients from healthy individuals and determine tumor cell proliferation and patient prognosis by mRNA levels. For example, NLGN3 and PTTG1 mRNA levels in serum exosomes of glioma patients are significantly higher than those of normal subjects, and the mRNA levels are higher, the worse the prognosis of patients [111]. MGMT mRNA in exosomes can predict the response of glioblastoma patients to antineoplastic drugs (temozolomide) [112], and some research teams have developed the exosome analysis platform iMER (based on EGFR/EGFRvIII exosome enrichment) and quantified its mRNA content in real time, so as to better determine the maximum efficiency of temozolomide treatment and/or how to select other treatment modalities in case of drug resistance, in addition, the use of iMER with auxiliary examination (such as MRI) can eliminate or reduce the need for biopsy in patient populations with the need of brain tumor and predict the best treatment regimen and prognosis [113]. At the same time, it has also been studied that mRNA-containing exosomes can restore the tumor inhibition function of orthotopically implanted PTEN-deficient brain gliomas, thereby inhibiting tumor growth and prolonging the survival of animals.

The current study shows exosomes represent an ideal treatment. Compared with viral carriers or nanoparticle carriers, the two most commonly used drug delivery systems, exosomes have a circulatory half-life, intrinsic ability to target tissues, biocompatibility, and minimal or no intrinsic toxicity problems. However, there are also some problems in exosome therapy, such as how to effectively incorporate high levels of mRNA into exosomes for targeted transcriptional manipulation and therapy. Although it has also been shown that exosomes containing endo-transcribed mRNA from a variety of cell sources can be obtained using cell nanopore (CNP technology) [101], the maturity, cost, feasibility of clinical application of the technology and the source of cells producing exosomes still need more intensified study. In addition, little is known about the key molecules of exosomes of the same or different cell sources under different conditions, and only by understanding the specific molecular mechanism and its regulatory network, can it be effectively applied in clinical practice. The exosome dose application method and effective exosome purification method also need further study.

## mRNA vaccine in the treatment of neural tumor

Direct vaccination with molecules encoding tumor-associated antigen messenger RNA (mRNA) is a novel and promising approach in cancer immunotherapy. With the continuous progress of research, mRNA vaccine is safe, efficient and relatively inexpensive, which makes it possible to be applied in cancer immunotherapy, and a breakthrough has been made in the treatment of glioblastoma (GBM) and other tumors. In recent years, due to the novel means of modifying mRNA in vitro, introducing it into the cytoplasm, expressing specific proteins, and achieving the purpose of immunotherapy, it has become a high-profile research topic.

Tumor growth and recurrence are associated with cancer stem cells, and glioblastoma stem cells (GSCs) can be isolated and expanded by spheroid formation assays [114, 115]. Vik-Mo et al. isolated brain tumor biopsies and prepared them into single cell suspension, amplified autologous cancer stem cells (CSCs) into tumor spheroids in vitro, amplified CSC mRNA, transfected monocyte-derived autologous dendritic cells (DCs), and treated the first seven patients with DC-based vaccines against cancer stem cells (CSCs) in solid tumors, and finally obtained 2.9-fold increase in progression-free survival in glioma patients [116].

It has been shown that cytomegalovirus (CMV) antigen is present in the basement membrane of 90% of GBM patients, but absent in normal brain [117–119]. This provides a rationale for using CMV antigens as tumor-specific immunotherapeutic targets. In addition, another recent clinical trial investigated adoptive immunotherapy with CMV phosphoprotein 65 (Pp65)-specific T cells in patients with recurrent GBM, and showed that 11 patients who received in vitro expanded CMV-specific T cells had a promising overall survival (OS) of 13.4 months and a progression-free survival (PFS) of approximately 8.1 months, and in vitro analysis of this study found no significant change in the polyfunctionality of CMV-specific T cells [120]. In this context, Elizabeth A et al. increased the frequency of multifunctional CMV pp65-specific CD8 + T cells after adoptive therapy with specific T cells by inoculating CMV pp65 RNA-loaded DCs, and randomized 17 patients into two groups: CMV pp65-specific T cells with either CMV-DC vaccine (CMV-ATCT-DC) or normal saline (CMV-ATCT-Saline) [121]. The results showed that the total frequency of interferon γ+, tumor necrosis factor α + and CCL3+ multifunctional and cytomegalovirus-specific CD8+ T cells was significantly increased in patients who received cytomegalovirus-ATCT-DC vaccination [122]. At present, based on the mRNA of T cells induced by tumor vaccine is relatively mature, the basic mechanism is to use the mRNA transfer the function of the genetic information of antigen presenting cell tumor related antigen can be converted to induction of immune responses, this method is relative to the obvious advantages of using DNA vaccine or peptides is mRNA itself does not directly affect the genome itself, and restrict human leukocyte antigen (HLA) [123].

In recent years, mRNA vaccine has been increasingly used in cancer research, showing a good application prospect. However, the relevant technical means still need to be improved. For example, the stability of extracted mRNA, the improvement of transfection rate, and the wide application in neural cell tumors, among others, still need our further study and discussion.

## Conclusion

### Summary and outlook

Initially, it was thought that RNA was a genetic material rather than DNA in organisms because mRNA was more active in cells relative to DNA, it had to carry codons frequently in and out of the nuclear pore, and it carried bases with a certain order, which was a behavior that was more conducive to the development and reproduction of organisms and was of great significance for biological existence. Until later, it was found that the genetic material of some viruses is not DNA, but RNA, and American scientists Termin and Baltimore found reverse transcriptase in viruses, which could reverse transcribe RNA into DNA [119]; thus, mRNA was shown to have a strong gene transcription function; later, it was found that RNA also had a regulatory function, largely dependent on RNA interference (RNAi) [124], and thus, the study of RNA-regulated proteins was widely carried out by scientists. In recent years, significant progress has been made in the cancer treatment of mRNA, of which, recent high-profile research topics have mainly focused on three aspects: 1. Chemical modification of mRNA, especially methylation modification; 2.Non-coding RNAs, especially lncRNAs, miRNAs, and circRNAs rich in exosomes, are widely studied because they are not found to affect the transcription of mRNAs; 3. mRNA tumor vaccines, have been drawing attention as a promising emerging tumor gene therapy.

mRNA is carried by the organism itself rather than serves as an invasive molecule, and its expression level can reflect the progression of the disease and serve as an important biomarker for diagnosis and prognosis. In addition, many malignant central nervous system tumors are primary tumors, and their pathogenesis is mostly due to gene mutations [88], so the use of mRNA to find cancer treatments can start from the source and play a role in gene therapy [125]. In recent years, in vitro transcription messenger RNA (IVT mRNA)-related technologies have been developed, and with the help of IVT mRNA, it can induce the de novo synthesis of desired proteins without changing the physiological status of target cells [126]; at the same time, mRNA delivery technologies are becoming more and more mature and diversified, and the way to deliver mRNAs using nanoparticles with substances such as liposomes [127] and exosomes [101] has become a high-profile research topic in recent years. Therefore, with the development of biomedical technology, the role of mRNA will be more and more effectively exerted in the treatment of neural tumors and even other types of tumors, and the investigation of the related research and function will also be deepened.

## Data Availability

Not applicable.

## Abbreviations

Nf1: Neurofibromin
mRNA: Messager RNA
siRNAs: Synthetic small interfering RNAs
RNAi: RNA interference
ICD-O: International Classification of Diseases
IDH-wt: IDH wild-type
IDH-mt: IDH mutant type
GBM: Glioblastoma
TAMs: Tumor-associated macrophages
IDH-wt: Diffuse astrocytomas, particularly wild-type
IGF2BP3: Insulin-like growth factor 2 mRNA-binding protein 3
WGCNA: Weighted co-expression network analysis
TGCT: Testicular germ cell tumors
esRNA: Exosome shuttle RNA
ATLL: Adult T-cell lymphoma/leukemia
MSC: Mesenchymal stem cells
RBPs: RNA-binding proteins
UTR: Untranslated region
MB: Medulloblastoma
RICH: Rapidly involuting congenital hemangiomas
NICH: Non-involuting congenital hemangiomas
PPI: Protein-protein interaction network
AAV: Adenovirus-associated vector
TLE1: Transcriptional co-pressor
Erbin: Erbb2-interacting protein
Nrg1: Neuregulin 1
CMV: Cytomegalovirus
OS: Overall survival
Pp65: Phosphoprotein 65
PFS: Progression-free survival
IVT mRNA: In vitro transcription messenger RNA
